# Malignant Melanoma With Neuroendocrine Differentiation: A Case Report and Literature Review

**DOI:** 10.3389/fonc.2021.763992

**Published:** 2021-12-01

**Authors:** Jason Cham, Ayal Shavit, Aren Ebrahimi, Miguel Viray, Paul Gibbs, Munveer S. Bhangoo

**Affiliations:** ^1^ Department of Internal Medicine, Scripps Clinic/Scripps Green Hospital, La Jolla, CA, United States; ^2^ Department of Pathology, Scripps Clinic/Scripps Memorial Hospital, Encinitas, Encinitas, CA, United States; ^3^ Department of Hematology and Oncology, Scripps Clinic/Scripps Green Hospital, La Jolla, CA, United States

**Keywords:** small cell carcinoma, malignant melanoma, neuroendocrine differentiation, checkpoint inhibitor, synaptophysin, genomics

## Abstract

**Background:**

Melanoma has a wide range of histologic variants and cytomorphologic features that make its diagnosis challenging. Melanoma can also rarely have neuroendocrine markers adding further diagnostic uncertainty particularly given that unrelated tumor types, such as prostate cancer, can also display focal neuroendocrine differentiations.

**Case presentation:**

Our patient is a 74-year-old Caucasian man found to have a lung mass. Initial biopsy revealed typical microscopic morphology and neuroendocrine differentiation consistent with small cell carcinoma. Despite standard chemoradiation treatment, the patient continued to progress with new metastasis in the brain, liver and bone. Subsequent chest wall biopsy revealed golden-brown pigment associated with melanin. Further tumor immunohistochemistry revealed extensive neuroendocrine differentiation with CD56, synaptophysin, and INSM1, as well as strong immunoreactivity for melanocyte markers including SOX10, S100, PRAME, and MITF, consistent with metastatic melanoma with neuroendocrine differentiation. Genomic testing revealed increased tumor mutational burden and alterations in NF1, BRAF, CDKN2A/B, TERT. The patient was transitioned to checkpoint inhibitor therapy with nivolumab and ipilimumab and had resolution of his intracranial mass and decrease in size of other metastatic lesions.

**Conclusion:**

Often the combination of anatomic findings such as a lung mass, typical microscopic morphology, and confirmation of neuroendocrine differentiation correctly identifies a patient with small cell carcinoma. However, in a patient who fails to respond to treatment, a broader immunohistochemical workup along with molecular testing with additional tissue may be warranted.

## Background

Small cell lung cancer (SCLC) is a neuroendocrine tumor that makes up 15 percent of all lung cancers. It is defined histologically as malignant epithelial tumor consisting of small cells with scant cytoplasm, ill-defined cell borders, finely granular nuclear chromatin, and absent of or inconspicuous nucleoli (1). Further immunohistochemistry including synaptophysin, chromogranin A, CD56, thyroid transcription factor 1 (TTF1), and MIB-1 can further help to confirm the diagnosis (1). Standard treatment includes combination chemotherapy with platinum-based therapy with etoposide. Immunotherapy with checkpoint inhibitors: durvalumab and atezolizumab have recently been shown to improve overall survival and progression free survival and have been added to standard therapy for extensive stage SCLC (1–3).

Melanoma has a wide range of histologic variants and cytomorphologic features. Additionally, melanoma can rarely display neuroendocrine differentiation further making its diagnosis challenging. Rare cases of melanoma with neuroendocrine differentiation have been reported, but most case reports describe the combination of immunohistochemical studies that led to the eventual diagnosis of malignant melanoma, but rarely describe the clinical management and outcome. In this case report, we present a patient who was initially diagnosed with small cell lung cancer based on tumor location, morphology and immunohistochemistry. We will discuss the course leading to the diagnosis of malignant melanoma with neuroendocrine differentiation including the genomic testing that helped to further guide clinical management.

## Case Presentation

A 74-year-old Caucasian man with a 20 pack-year smoking history but no significant past medical history presented with chest pain and shortness of breath found to have an non-ST elevation myocardial infacrction requiring percutaneous coronary intervention with placement of five bare metal stents. He was incidentally found to have a 3.5 cm right lower lobe mass on chest radiography. He underwent PET-FDG which confirmed a 2.2 cm right lower lobe mass without evidence of metastases. The patient then discussed a right lower lobectomy with lymph node dissection with cardiothoracic surgery. However, because he was recently status post placement of five coronary artery stents and would need to continue antiplatelet therapy, decision was made to delay the intervention. Observation 3 months later with CT-imaging revealed that the mass had enlarged to 3.4 cm, now with hilar and mediastinal lymphadenopathy.

**Figure 1:**
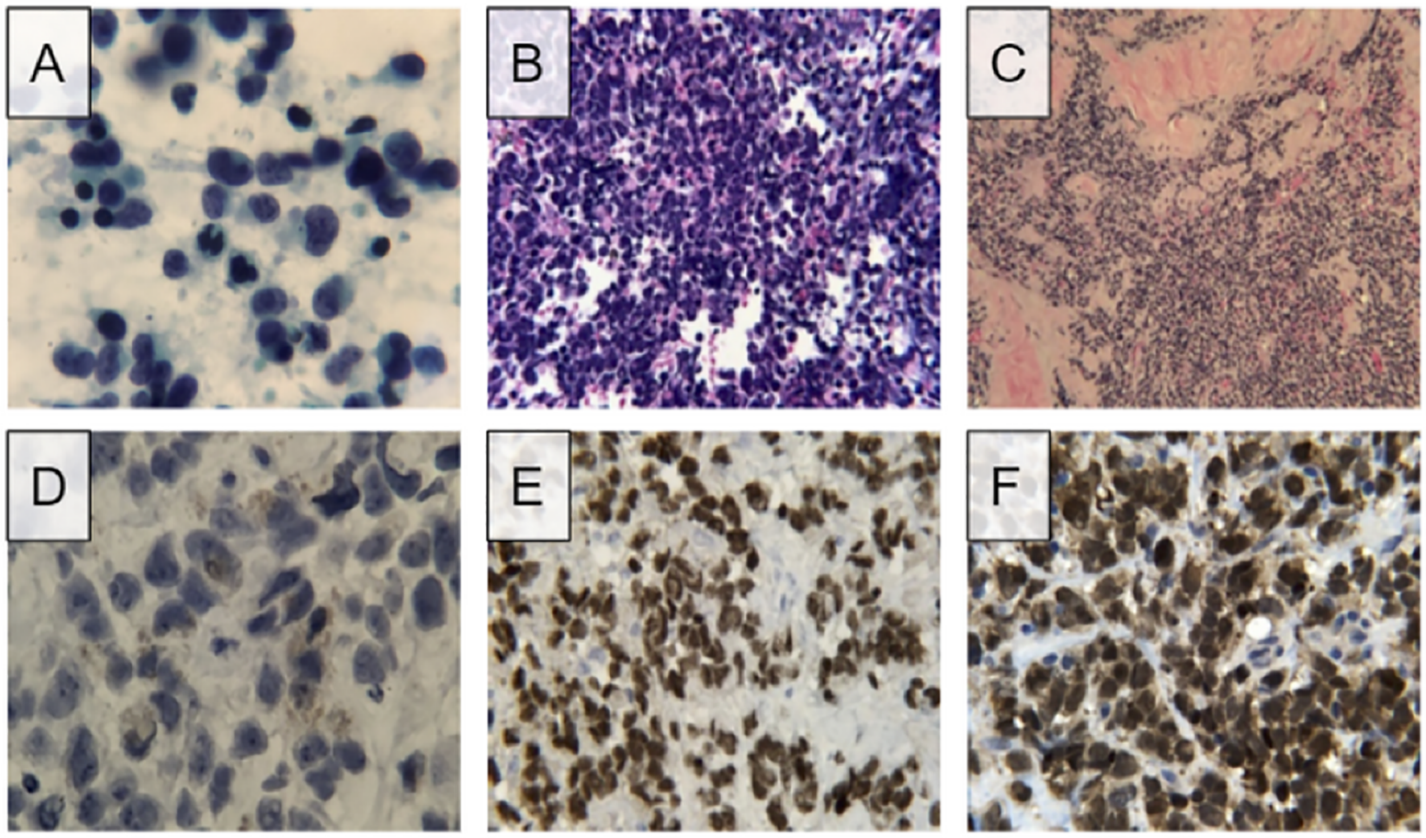
Microscopic findings and immunohistochemistry stains of initial lymph node FNA and subsequent needle-core biopsy. **(A)** Direct smear of lymph node FNA shows pleomorphic cells with scant sytoplasm and fine chromatin, presence of nucleoli was not detected. **(B)** Routine hematoxylin and eosin staining of cell block visualizes numerous small cell populations with high nuclear to cytoplasmic ratio. **(C)** Routine hematoxylin and eosin staining of needle-core biopsy shows populations of small pleomorphic cells. **(D)** High magnification image of needle-core biopsy displays golden-brown coloration resembling that of melanin. **(E)** Positive result for SOX10 staining, a biomarker for melanoma. **(F)** Positive results for S100 staining, another biomarker for melanoma.

The patient subsequently underwent endobronchial biopsy of mediastinal lymph nodes. The initial specimen, derived from station 7 and 12R lymph nodes, showed the following morphologic findings on direct smear preparations and cell block sections: abundant foci of small malignant cells demonstrating pleomorphism and high nuclear to cytoplasmic ratios. The cells displayed scant cytoplasm and finely granular chromatin along with irregular nuclear membranes and infrequent nuclear molding. Nucleoli were inconspicuous or absent (**Figures 1A, B**). Immunohistochemical studies were further performed on the cell block material. The immunohistochemical analysis was carried out in a routine pathology laboratory setting with clinically accredited antibodies and standardized methodology. It demonstrated tumor cell reactivity with antibodies to synaptophysin and CD56, and no significant reactivity with antibodies to cytokeratin AE1/AE3, TTF-1, chromogranin A, p40, and CD45. The Ki-67 index was 90-100%. Flow cytometry results were negative for lymphoproliferative processes. Thus, the histopathology was consistent with small cell carcinoma presumably of lung origin.

**Figure 2:**
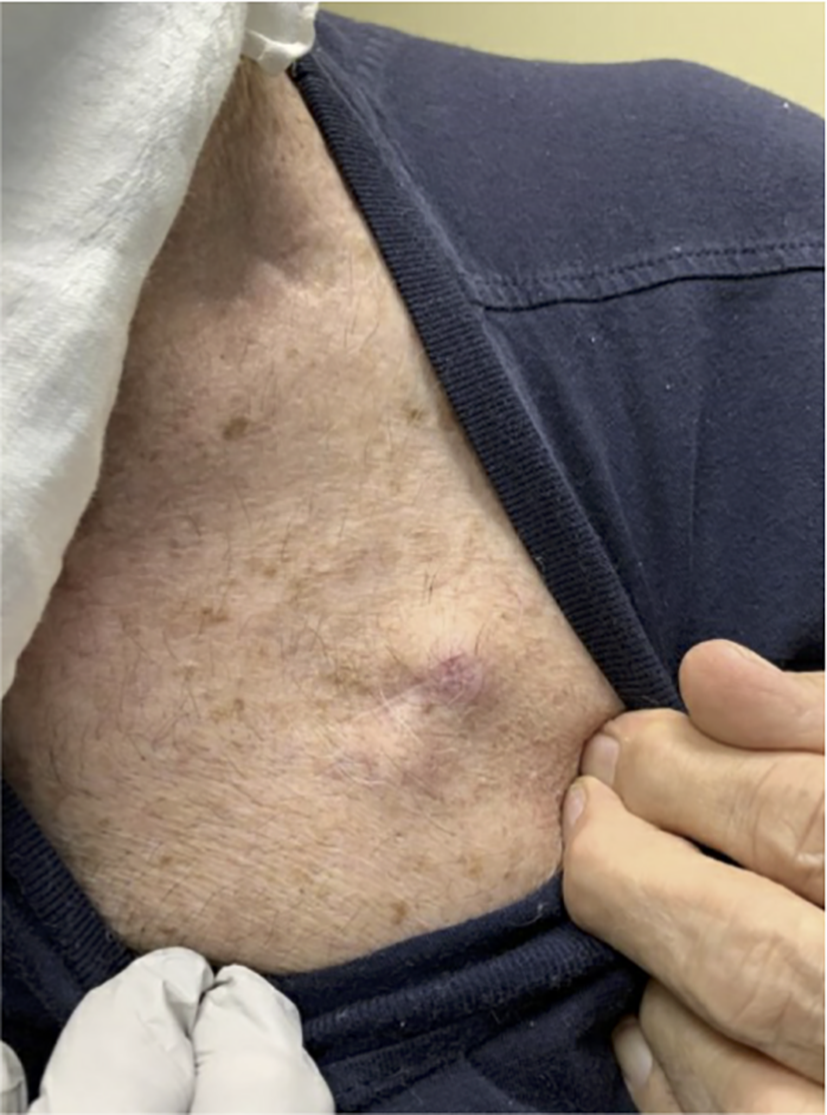
Image of left chest wall lesion.

He initiated chemoradiation with 4 cycles of cisplatin and etoposide with radiation to the right lower lobe and right hilum. Checkpoint inhibition was not included as this was still considered limited stage SCLC. Unfortunately, 2 months after completing chemoradiation therapy, MRI of the brain revealed new solitary right frontal lobe metastases. PET-FDG showed hepatic and bone metastases. Additional areas of metastatic progression included mediastinal/retroperitoneal lymph nodes and lung. Clinical dermatologic examination revealed a new chest wall lesion (**Figure 2**).

Biopsy of the chest wall lesion was performed which showed malignant cells extensively invading into adipose and connective tissue. Similar to the previous biopsy, these cells had scant cytoplasm, fine nuclear chromatin, and pleomorphic nuclei were observed. Small nucleoli were also visible. This biopsy is primarily distinguished from the previous lymph node samples by the presence of myxoid stromal change and the prominence of foci with a golden-brown pigment associated with melanin. The unique coloration of the cells supported a possible diagnosis of malignant melanoma, as opposed to the initial diagnosis of small cell carcinoma.

Immunohistological testing revealed tumor cell reactivity with antibodies to synaptophysin and CD56, and no significant reactivity with antibodies to cytokeratin AE1/AE3, TTF-1, chromogranin A, p40, and CD45. The Ki-67 index was 90-100%. Given the overlapping but somewhat distinct appearance of this sample, immunohistochemical studies were again performed, this time demonstrating positive tumor reactivity to antibodies for synaptophysin, S100, SOX10, and FLI1. Focal positivity for MITF was also observed. No tumor cell reactivity was observed with cytokeratin AE1/AE3, TTF-1, chromogranin A, CD45, OSCAR, EMA, CK7, CK20, MART-14, HMB45, neurofilament, desmin, h-caldesmon, myogenin, CD34, or CD99. Further immunohistochemical studies revealed positivity for PRAME, focal positivity for INSM1, and no reactivity with BRAF V600E, NRAS, Q61R, or c-kt. Additional tests on the initial lymph node biopsy were also performed, exhibiting positivity for S100 and SOX10. Review of the initial material, including close examination of the cell cytoplasm on negative control slides stained with hematoxylin only, allowed recognition of a small percentage of cells exhibiting fine, brown pigmented material in the cytoplasm, compatible with melanin (**Figures 1C–F**).

PD-L1 (Dako 22C3 pharmDx) tumor proportion score (TPS) was 0%. FoundationONE CDx (F1CDx) comprehensive genomic profiling of the chest wall lesion revealed an elevated tumor mutational burden (TMB) of 21 mutations per megabase with stable microsatellite status. Additional genomic alterations were detected including: BRAF G469E, loss of CDKN2A and CDKN2B, HGF amplification, NF1, PALB2, and TERT promoter. Based upon the combination of morphologic and immunohistochemical findings in the two specimens as well as genetic profiling, a diagnosis of malignant melanoma with neuroendocrine differentiation was made.

Given this new diagnosis of malignant melanoma with neuroendocrine differentiation, the patient was started on nivolumab 1mg/kg and ipilimumab 3mg/kg q3W for three cycles. The patient experienced Grade 3 liver function testing (LFT) elevation and the fourth cycle of ipilimumab was omitted. The patient’s LFT abnormalities resolved with a short course of glucocorticoids and he was continued on nivolumab maintenance (480mg IV q4W).

**Figure 3:**
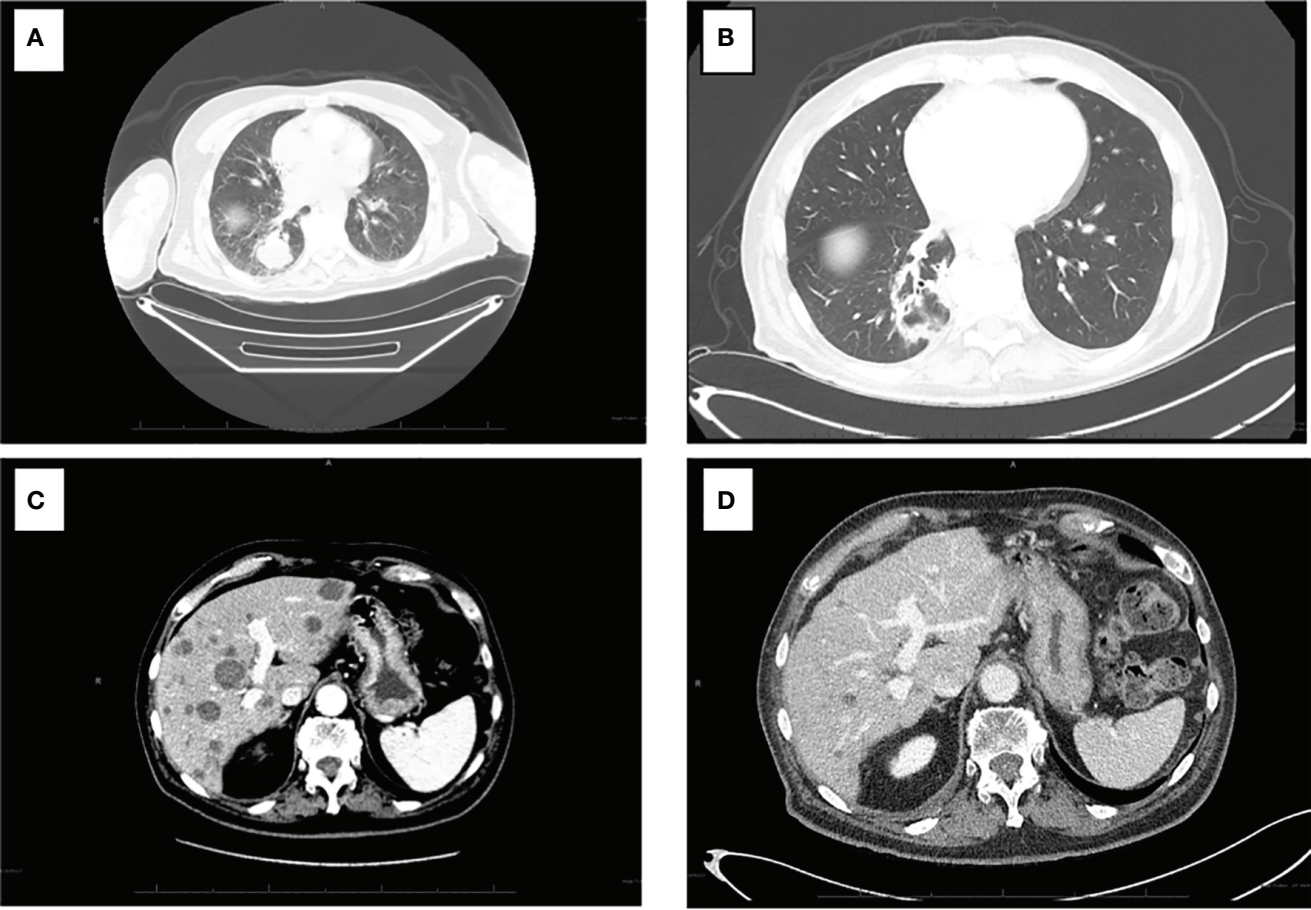
CT of the chest and abdomen pre and post treatment with combination nibolumab and ipiliimumab. **(A)** Intrathoracic lymphadenopathy anf right lower lobe lung nodule. **(B)** Interval resolution of intrathoracic lymphadenopathy and right lower lung nodule. **(C)** Numerous hepatic metastasis and retroperitoneal lymphadenopathy with the largest hepatic lesion measuring 3.7 cm. **(D)** Resolution of retroperitoneal lymphadenopathy and marked interval decrease size of hepatic metastasis with lesion now measuring 1.7 cm.

Interval reimaging has demonstrated marked and durable radiographic response with significant reduction in disease burden. The previously noted innumerable hypoenhancing metastatic lesions in the liver has all decreased in size with the largest now measuring 1.7 cm (baseline 3.7 cm). The patient’s previously noted intrathoracic and retroperitoneal lymphadenopathy as well as right lower lung nodule have resolved. No further progression in osseous metastasis was noted with stability in previously identified sclerotic lesions involving right humeral head, right L4, left S1, and right ilium. (**Figures 3A–D**).

## Methods

We conducted a systematic review to identify the patterns of immunohistochemical (IHC) findings and pitfalls commonly encountered in the diagnosis of melanomas with neuroendocrine differentiation. A literature search was conducted in the PubMed database using the search terms “Melanoma with neuroendocrine differentiation”. 194 publications resulted as of June 20, 2021. All 194 articles were reviewed and 9 were relevant to this review. Of the 9 total publications reviewed, 12 cases of melanoma with neuroendocrine differentiation were identified. 11 had the diagnosis of melanoma with neuroendocrine differentiation initially and 1 had an initial diagnosis of another malignancy that was later corrected to melanoma with neuroendocrine differentiation after further pathological processing. 17 IHC cases were reported in total.

## Discussion

Melanoma has a wide range of cytomorphologic and histologic variants that make its diagnosis challenging. Here, we report a patient who was originally diagnosed with small cell carcinoma based on the biopsy morphology, neuroendocrine marker expression and clinical presentation. Lack of keratin and TTF-1 expression can rarely occur in small cell carcinoma but does not exclude the diagnosis. Unlike small cell carcinoma which does not express markers of melanocytic differentiation, malignant melanoma can rarely exhibit neuroendocrine differentiation. This case demonstrates the plasticity of malignant melanoma in that it shows extensive neuroendocrine differentiation with CD56, synaptophysin, and INSM1. Strong immunoreactivity for multiple melanocytic markers including SOX10, S100, PRAME, MITF and retained nuclear RB expression support the final diagnosis of malignant melanoma rather than small cell carcinoma.

In the largest case series to date, Romano et al. report 73 patients with malignant melanoma and aberrant expression of various tumor markers on immunohistochemistry (4). Synaptophysin expression was reported at 29% (10/34 cases), while our review of the literature revealed 56% positivity in 9/16 reported IHC results. Neurofilament protein expression was reported at 16% (5/31 cases), as compared to 83% positivity in 10/12 IHC results found in our literature review (5–10). Of note, Romano et al. reported that chromogranin expression was absent in all 32 cases they investigated that were stained for chromogranin. This is consistent with our case, which was also negative for chromogranin, however, of the 16 IHC results with chromogranin staining reviewed in the literature, 10 (63%) did demonstrate positivity for chromogranin. CD56 and INSM1 staining was not reported by Romano et al, but they were seen with 100% positivity, in 7 and 1 IHC staining results, respectively, that we reviewed as well as in our reported case (**Table 1**) (4–10).

**Table 1:** Summary of Reported Melanoma Cases with Neuroendocrine Differentiation.

Case report		Melan-A	SOX10	S100	HMB45	Tyrosinase	Chromogranin	SYP	CD56	INSM1	NF	Treatment	Outcome
Juhlin et al., (5)	Case 1: Chest Wall	-	ND	ND	-	ND	-	+	+	+	ND	Carboplatin, Etoposide	Death
	Case 1: Femur	+	+	ND	+	ND	-	+	+	ND	ND	Carboplatin, Etoposide	Death
Eyden et al., (6)	Case 1: Cutaneous lesion	ND	ND	ND	ND	ND	ND	ND	ND	ND	ND	NR	NR
	Case 1: Axillary Mass	+	ND	+	+	+	+	+	+	ND	+	NR	NR
	Case 2: Nasal Mucosal lesion	+	ND	+	+	+	+	+	+	ND	+	NR	NR
	Case 2: Cervical lymph node	+	ND	+	+	+	+	+	+	ND	+	NR	NR
	Case 3: Cutaneous lesion	+	ND	+	+	+	-	-	ND	ND	-	NR	NR
	Case 3: Axillary lymph node	+	ND	+	+	ND	+	+	ND	ND	+	NR	NR
	Case 3: Pulmonary metastasis	-	ND	+	-	-	+	-	ND	ND	-	NR	NR
Katerji et al., (7)	Case 1: Esophageal mass	+	+	+	ND	ND	-	-	+	ND	ND	Pembrolizumab	Desease progression and death
Lee et al., (11)	Case 1: paranasal sinus	+	ND	+	+	ND	+	-	ND	ND	+	NR	Liver matastasis
	Case 2: maxillary sinus and nasal cavity	+	ND	-	+	ND	-	-	ND	ND	+	NR	Local recurrence
	Case 3: nasal cavity	+	ND	+	+	ND	+	+	ND	ND	+	NR	Metastasis to lymph node
	Case 4: nasal cavity, ethmoid, and sphenoid sinus	+	ND	+	+	ND	+	-	ND	ND	+	NR	Local recurrence and metastasis to lymph node
	Case 5: nasopharyngeal space	+	ND	+	+	ND	+	-	ND	ND	+	NR	Local recurrence and tracheal metastasis

ND, Not Done; NR, Not Reported.

When combining the above findings of neuroendocrine marker expressivity to expression of melanocytic markers on immunohistochemistry, the challenges in diagnosis become more apparent. This is due to the fact that malignant melanomas with aberrant protein expression do not present with complete or near complete expressivity of all immunohistochemical melanoma markers. Romano et al. report positivity for S100 at 92% (60/65 cases), Melan-A at 53% (34/64), HMB45 at 50% (30/60), and Tyrosinase at 40% (16/40) (4). In comparison, the IHC results from our review of the literature for cases of melanoma with neuroendocrine differentiation revealed positivity for S100 at 92% (12/13), Melan-A at 87% (13/15), HMB45 at 87% (13/15) and Tyrosinase at 80% (4/5). SOX10 positivity in our literature review was also seen at 100% (2/2) (**Table 1**) (4–10).

Previous descriptions of malignant melanoma with neuroendocrine differentiation have emphasized the diagnostic pitfalls posed by such a tumor, which may present in anatomic locations which are statistically more likely to harbor a neuroendocrine carcinoma than a melanocytic tumor (5). With the discovery of novel tumor markers and the development of advanced techniques in immunohistochemical staining, non-melanocytic markers in melanoma have been increasingly identified and the overlap in immunohistochemical findings is significant.

Advances in genomics and molecular testing have added an additional layer of diagnostic testing to guide therapeutic choices. Our patient’s profiling returned with multiple genomic mutations, two of which were an increased tumor mutational burden (TMB) and having a stable microsatellite status (MS-Stable). TMB is a measure of the number of somatic protein coding base substitution and insertion/deletion mutations occurring in a tumor specimen which can be seen in various melanoma subtypes with TMB ranging from 6.3-14.4 Muts/Mb (12). Of note, our patient’s TMB was 21 Muts/Mb which is higher than average. Previous studies have shown that increased TMB’s have been associated with longer progression free survival (PFS) and overall survival (OS) for patients with melanoma treated with nivolumab (13). MS status has also been studied with regard to immunotherapy sensitivity, with MS-Stable profiles being associated with less response to anti-PD-1 immune checkpoint inhibitors compared to MS-Instability (MS-I) profiles (14–16). TMB and MS status are therefore highly important and serve as a genomic blueprint in both detection and treatment options for melanoma.

Additional genomic markers in the patient’s genomic profiling included alterations in NF1 (reported in 13% of all melanoma cases) (17), BRAF (41-51% of cutaneous melanoma cases) (18), CDKN2A/B (14-29% of all melanoma cases) (19) and TERT (22-71% of all melanoma cases) (20). Each of these mutations confers additional profiling of the tumor, assisting in diagnosis when immunohistochemical findings are inconclusive. Unfortunately, our patient had a non-canonical mutation BRAF that was not amenable to targeted therapy. However, overall, these genomic markers involved in multiple cell cycle pathways may be an improved method for identifying tumor origin and subsequent treatment options, especially in cases such as ours where initial tissue biopsy can be misleading.

The current example serves as a cautionary tale in which the recommended heuristic method of imputing a diagnosis of lung carcinoma is defeated by a rare entity. In the great majority of patients, the combination of anatomic findings (a lung mass), typical microscopic morphology, and confirmation of neuroendocrine differentiation correctly identifies a patient with small cell carcinoma. Although clinically efficacious, this heuristic is fragile in that it breaks down when a greater volume of tissue would be informative and prompt a broader immunohistochemical and genomic workup, and that it potentially overemphasizes the significance of the anatomic site in which the tumor is first detected, given that melanomas occur capriciously in many body sites other than skin. As such, we believe that when a clinician encounters a similar malignancy with neuroendocrine features or a neuroendocrine tumor of unknown primary, consideration should be given for malignant melanoma, particularly if the patient does not improve with initial therapy. Positive immunohistochemical staining for neuroendocrine markers does not exclude the diagnosis of malignant melanoma. Further tissue processing and staining for melanocytic markers, such as Melan-A and S100, should be included. Subsequent genomic testing particularly for TMB, NF1, BRAF, CDKN2A/B, TERT can facilitate the diagnosis and guide future management. These steps are critical for the identification of a malignant melanoma with neuroendocrine differentiation, particularly as the treatment for melanoma is different from the treatment for neuroendocrine tumors and can lead to significant clinical outcomes.

## Data Availability Statement

The original contributions presented in the study are included in the article/supplementary material. Further inquiries can be directed to the corresponding author.

## Ethics Statement

Written informed consent was obtained from the individual(s) for the publication of any potentially identifiable images or data included in this article.

## Author Contributions

JC took the lead in the writing of the manuscript. MB was the primary provider. MV and PG reviewed and prepared figures for the histopathologic findings. JC and AE performed the majority of literature review. AS, AE, MV, PG, MB reviewed, wrote, and helped edit the manuscript. All authors contributed to the article and approved the submitted version.

## Funding

JC was supported by the National Center for Advancing Translational Sciences, National Institutes of Health, through grant number UL1TR002550 and linked award KL2TR002552.

## Author Disclaimer

The content is solely the responsibility of the authors and does not necessarily represent the official views of the NIH.

## Conflict of Interest

The authors declare that the research was conducted in the absence of any commercial or financial relationships that could be construed as a potential conflict of interest.

## Publisher’s Note

All claims expressed in this article are solely those of the authors and do not necessarily represent those of their affiliated organizations, or those of the publisher, the editors and the reviewers. Any product that may be evaluated in this article, or claim that may be made by its manufacturer, is not guaranteed or endorsed by the publisher.

## Abbreviation

PET-FDG, Positron Emission Tomography-Fluorodeoxyglucose; CT, Computed Tomography; MRI, Magnetic Resonance Imaging; LFT, Liver function test.
